# Supplementary material for: Convergent evidence for the molecular basis of musical traits
Source: Sci Rep. 2016 Dec 22;6:39707. doi: 10.1038/srep39707 (PMC5177873; doi:10.1038/srep39707)
Supplement: Supplementary Tables and Figures [file srep39707-s1.pdf]

Supplementary Information

Convergent evidence for the molecular basis of musical traits

Jaana Oikkonen[1,2]*, Päivi Onkamo[2], Irma Järvelä[1], Chakravarthi Kanduri[1]

(1) Department of Medical Genetics, University of Helsinki, P.O. Box 720, 00014 University of Helsinki, Finland

(2) Department of Biosciences, University of Helsinki, P.O. Box 56, 00014 University of Helsinki, Finland

*Corresponding author: Jaana Oikkonen, University of Helsinki, P.O. Box 56, 00014 University of Helsinki, FINLAND; Email: jaana.oikkonen@helsinki.fi

Table S1: Human studies used for the convergent analysis.

Article	DNA<br><br>Candidate /GW	RNA<br><br>Candidate /GW	Protein / hormone Type	Phenotype (music-related)
Granot et al. 2007	Candidate	-	-	Musical ability (tests)
Theusch et al. 2009	GW	-	-	Absolute pitch
Park et al. 2012	GW	-	-	Singing pitch accuracy
Morley et al 2012	Candidate	-	-	Choir participation
Gregersen et al. 2013	GW	-	-	Absolute pitch and synesthesia
Fukui and Toyoshima 2013	Candidate	-	-	Music listening and emotions
Oikkonen et al. 2015	GW	-	-	Musical aptitude (tests)
Ukkola et al. 2009	Candidate	-	-	Musical aptitude (tests)
Liu et al. 2016	GW	-	-	Musical aptitude (tests)
Emanuele et al. 2009	-	Candidate	-	Musicians
Qu et al. 2013	-	GW	-	Music listening
Kanduri, Kuusi et al. 2015	-	GW	-	Playing music
Kanduri, Raijas et al. 2016	-	GW	-	Listening music
Hassler et al. 1992	-	-	Levels	Music composition, musical ability
Bartlett et al. 1993	-	-	Levels	Music listening
VanderArk and Ely 1993	-	-	Levels	Music listening
Möckel et al. 1995	-	-	Levels	Music listening
McCraty et al. 1996	-	-	Levels	Music listening
Gerra et al. 1998	-	-	Levels	Music listening
Fukui and Yamashita 2003	-	-	Levels	Music listening
Khalfa et al. 2003	-	-	Levels	Music listening
Stefano et al. 2004	-	-	Levels	Music listening
Kreutz et al. 2004	-	-	Levels	Choir singing and song listening
Nilsson 2009	-	-	Levels	Music listening
Lai and Li 2011	-	-	Levels	Music listening
Salimpoor et al. 2011	-	-	Brain release	Music listening
Thoma et al. 2013	-	-	Levels	Music listening
Granot et al. 2013	-	-	Administration	Musical ability (tests)
Gingras et al. 2014	-	-	Levels	Music listening
Schwilling et al. 2015	-	-	Levels	Music listening
Fancourt et al. 2015	-	-	Levels	Singing
Keeler et al. 2015	-	-	Levels	Vocal improvisation
Fancourt and Williamon 2016	-	-	Levels	Attending concert
Gervain et al. 2013	-	-	Administration	AP task and test
Knight and Rickard 2001	-	-	Levels	Music listening

Table S2: Songbird studies used for the convergent analysis

Article	Songbird	Phenotype	Core molecule data
Leblois et al. 2012	Zebra finch	Singing: undirected and directed	Dopamine D1 antagonist
Jarvis et al. 1998	Zebra finch	Singing: directed or undirected	RNA: EGR1 expression
Jansen et al. 2005	Zebra finch	Singing and song length	Mel1B antagonist and melatonin
Rauceo et al. 2008	Zebra finch	Singing: directed	Dopamine receptor antagonists
Olson et al. 2015	Zebra finch	Sensitivity period	RNA: candidate gene expression
Mello et al. 1992	Canary and zebra finch	Song listening	RNA: EGR1 expression
Velho et al. 2008	Zebra finch	Song listening	RNA: Syn2 and Syn3 expression
London and Clayton 2008	Zebra finch	Song learning; Sensitivity period	Inhibition of MEK
Gunaratne et al. 2011	Zebra finch	Song listening	RNA: miRNA sequencing
Teramitsu and White 2006	Zebra finch	Singing: undirected and directed	RNA: FOXP2 and EGR1 expression
Teramitsu et al. 2010	Zebra finch	Sensorimotor learning	RNA: FOXP2 expression
Haesler et al. 2004	Songbirds versus non-songbirds	Vocal learning and vocal plasticity	RNA: FOXP2 and FOXP1 expression
Haesler et al. 2007	Zebra finch	Vocal imitation	Inhibition of FOXP2
Miller et al. 2008	Zebra finch	Singing: undirected and directed	Protein: FOXP2 expression
Dong and Clayton 2008	Zebra finch	Listening conspecific song	MEK inhibition and EGR1 expression
Basham et al. 1996	Zebra finch	Sing learning	Antagonist for NMDA
Wada et al. 2006	Zebra finch	Singing: undirected and directed	RNA: microarray expression
Matsunaga et al. 2011	Three different songbirds	Music learning during sensitive period	RNA: NR3C2 expression
Hartog et al. 2009	Canary	Singing	Manipulations and EGR1 expression
Mori and Wada 2015	Zebra finch	Vocal plasticity	RNA: GW expression and testosterone
Yoder et al. 2015	Zebra finch	Vocal memory	Estradiol administration
Abe et al. 2015	Zebra finch	Song learning and audio memory	Transgenic
Hilliard et al. 2012	Zebra finch	Singing: undirected	Microarray RNA expression, candidate protein expression
Warren et al. 2010	Zebra finch	Singing behaviour	RNA: microarray gene expression
Avey et al. 2008	Black-capped chickadee	Singing and listening birdsong	EGR1 protein expression
Whitney et al. 2014	Zebra finch	Singing for 0.5-7 hrs vs silence.	RNA: microarray expression
Bailey and Wade 2003	Zebra finch	Auditory stimulation; sensitivity period	FOS and EGR1 protein expression
Eda-Fujiwara et al. 2003	Budgerigar	Song listening: complexity	EGR1 protein expression
Pinaud et al. 2008	Zebra finch	Listening conspecific song	Proteomics
Poopatanapong et al. 2006	Zebra finch	Singing: undirected and directed	RNA: expression of EGR1, Syt I, IV
Shi et al. 2013	Zebra finch	Undirected singing; song development	RNA: candidate miRNA expression
Heston and White 2015	Zebra finch	Undirected singing; song imitation	FOXP2 overexpression

Sasaki et al. 2006	Zebra finch	Singing: undirected and directed	Dopamine reuptake inhibitor
Bolhuis et al. 2000	Zebra finch	Song memory	FOS and EGR1 protein expression
Drnevich et al. 2012	6 species	Song playback	RNA microarray expression
Lombardino et al. 2005	Zebra finches	Directed singing	RNA: microarray expression
Nastiuk et al. 1994	Zebra finch and canary	Song playback	RNA: EGR1 and c-jun expression
Miller et al. 2015	Zebra finch	Undirected and directed singing; vocal variability	Dopamine inhibition and candidate protein expression
Thompson et al. 2013	Zebra finch	Singing: directed or undirected	RNA: FOXP2 expression
Tremere et al. 2009	Zebra finch	Song listening	Manipulations and candidate gene expressions
Huesmann and Clayton 2006	Zebra finch	Song listening	Inhibition and candidate protein and EGR1 RNA expression
DeVries et al. 2015	Starling	Singing: directed	RNA: DRD1 and DRD2 expression
Fusani et al. 2003	Canary	Singing	Hormone levels and candidate gene expression
Gilbert et al. 2013	Zebra finch	Song listening	Antagonist and protein expression
Murugan et al. 2013	Zebra finch	Singing: undirected and directed; song learning	Knockdown and protein expression
Merullo et al. 2015	Starling	Directed singing	RNA: NT and dNTSR1 expression
Monbureau et al. 2015	Canary	Song listening	Protein: c-fos expression
Chen et al. 2013	Bengalese and zebra finch	Singing: undirected	RNA: FOXP2 and FOXP1 expression
LeBlanc et al. 2007	White-throated sparrows	Song listening	Hormone and protein expression
Lynch et al. 2012	Zebra finch	Song listening	TH and EGR1 protein expression
Kimpo et al. 1997	Zebra finch	Singing	FOS protein expression
Velho et al. 2005	Zebra finch	Song listening; Singing: undirected and directed	MEK inhibition and candidate gene RNA expression
Singh et al. 2000	Zebra finch	Song learning; Sensitivity period	Testosterone manipulation and NR1, NR2B gene expression
Velho et al. 2012	Zebra finch	Song listening	Antagonist, candidate gene and protein expression
Riters et al. 2014	European Starling	Singing: undirected	RNA: candidate gene expression

Table S3: Other animals' studies used for the convergent analysis

Article	Animal	Phenotype	Study type
Arnauld et al. 1996	Mouse	Auditory stimulation with musical sounds	RNA: c-fos expression
Sutoo et al. 2004	Rat	Music exposure	Dopamine brain expression
Shu et al. 2005	Mouse	Ultrasonic vocalization	Knockout of FOXP2
Chikahisa et al. 2006	Mouse	Music exposure: perinatal period and postnatal	Candidate gene protein expression
Angelucci et al. 2007	Mouse	Music exposure	Candidate gene protein expression
Xu et al. 2009	Rat	Music listening	NR2B protein expression
Mangiamele et al. 2008	Tungara frogs	Listening conspecific call	RNA: EGR1 expression
Meng et al. 2009	Mouse	Music exposure	RNA: microarray expression
Kurz et al. 2010	Mouse	Ultrasonic vocalization	Knockout and microarray RNA expression
Kurz et al. 2011	Mouse	Ultrasonic vocalization	Knockout and microarray RNA expression
Ringel et al. 2013	Rat	Ultrasonic vocalization towards female	Dopamine receptor antagonists
Sia et al. 2013	Mouse	Vocalization	SRPX2 inhibition and protein expression
Sanyal et al. 2013	Chicken	Music exposure	Hormone levels and protein expression
Chaudhury et al. 2009	Chicken	Music exposure	Protein expression
Yang 2012	Mouse	Music preference, critical period	Valproate injection, NgR knockout and c-fos expression

Table S4: Functional enrichment of the top 40 candidate genes, in the order of significance.

Diseases or functions annotation	p-value	Molecules / top 40 molecules	# / top 40 molecules
Cognition	1.69E-16	ARC, AVPR1A, BDNF, estradiol, dopamine, DOPEY2, DRD1, EGR1, FOS, GRIN2B, cortisol, MAPK10, noradrenalin, NR4A2, NTRK2, POMC, SNCA	16
Memory	2.00E-14	ARC, AVPR1A, BDNF, estradiol, dopamine, DRD1, EGR1, GRIN2B, cortisol, noradrenalin, NTRK2, POMC, SNCA	14
Learning	2.54E-14	ARC, AVPR1A, BDNF, estradiol, dopamine, DRD1, EGR1, FOS, GRIN2B, cortisol, noradrenalin, NR4A2, NTRK2, POMC, SNCA	12
Excitation of neurons	5.40E-12	BDNF, dopamine, DRD1, FOS, noradrenalin, NTRK2, RBFOX1, SNCA	8
quantity of catecholamine	3.12E-11	BDNF, estradiol, dopamine, cortisol, MAPK10, noradrenalin, NR4A2, POMC, SNCA	9
apoptosis of brain	6.16E-11	BDNF, estradiol, dopamine, DUSP1, EGR1, FOS, GRIN2B, MAPK10, NTRK2	9
epilepsy	7.91E-11	ARC, BDNF, DUSP1, DUSP5, EGR1, FOS, GRIN2B, MAPK10, NR4A3, NTRK2, RBFOX1	10
behavior	1.24E-10	ARC, AVPR1A, BDNF, estradiol, dopamine, DRD1, DUSP1, EGR1, FOS, GRIN2B, cortisol, noradrenalin, NR4A2, NR4A3, NTRK2, POMC, SNCA	8
transcription	1.63E-10	ARID1B, BDNF, estradiol, dopamine, DRD1, DUSP1, DUSP5, EGR1, FOS, FOSL2, FOXP2, cortisol, MAPK10, noradrenalin, NR4A2, NR4A3, NTRK2, PHIP, PKIA, POMC, SNCA, TET2	12
transcription of RNA	1.90E-10	ARID1B, BDNF, estradiol, dopamine, DRD1, DUSP1, DUSP5, EGR1, FOS, FOSL2, FOXP2, cortisol, MAPK10, noradrenalin, NR4A2, NR4A3, PHIP, PKIA, POMC, SNCA, TET2	21
quantity of cells	2.07E-10	AVPR1A, BDNF, estradiol, dopamine, DRD1, DUSP1, EGR1, FOS, FOSL2, GRIN2B, HLA-A, cortisol, IRS2, MAPK10, noradrenalin, NR4A2, NR4A3, NTRK2, POMC, SNCA, TET2	22
apoptosis of striatal neurons	2.41E-10	BDNF, dopamine, DUSP1, GRIN2B, NTRK2	21
apoptosis of neurons	2.49E-10	BDNF, estradiol, dopamine, DUSP1, EGR1, FOS, GRIN2B, MAPK10, NR4A2, NR4A3, NTRK2, SNCA	7
long-term potentiation of brain	3.29E-10	BDNF, estradiol, dopamine, DRD1, EGR1, GRIN2B, NTRK2, SNCA	10
long-term potentiation	3.63E-10	ARC, BDNF, estradiol, dopamine, DRD1, EGR1, GRIN2B, noradrenalin, NTRK2, SNCA	10
diabetes mellitus	4.17E-10	BDNF, estradiol, DRD1, DUSP1, EGR1, FOS, GRIN2B, HLA-A, cortisol, IRS2, MAPK10, noradrenalin, NR4A2, NR4A3, NTRK2, RBFOX1, SNCA	11
synthesis of D-glucose	5.42E-10	AVPR1A, cortisol, IRS2, noradrenalin, NR4A2, NR4A3, POMC	8
expression of RNA	6.86E-10	ARID1B, BDNF, estradiol, dopamine, DRD1, DUSP1, DUSP5, EGR1, FOS, FOSL2, FOXP2, cortisol, MAPK10, noradrenalin, NR4A2, NR4A3, NTRK2, PHIP, PKIA, POMC, SNCA, TET2	16
cell death of brain cells	9.01E-10	BDNF, estradiol, dopamine, DUSP1, EGR1, FOS, GRIN2B, MAPK10, NTRK2, SNCA	22
glucose metabolism disorder	9.58E-10	BDNF, estradiol, DRD1, DUSP1, EGR1, FOS, GRIN2B, HLA-A, cortisol, IRS2, MAPK10, noradrenalin, NR4A2, NR4A3, NTRK2, POMC, RBFOX1, SNCA	10
epileptic seizure	1.02E-09	ARC, BDNF, DUSP1, DUSP5, EGR1, FOS, MAPK10, NR4A3	7
feeding	1.07E-09	BDNF, estradiol, dopamine, DRD1, DUSP1, GRIN2B, noradrenalin, NR4A2, NTRK2, POMC	14
seizures	1.09E-09	ARC, BDNF, DUSP1, DUSP5, EGR1, FOS, GRIN2B, MAPK10, NR4A3, NTRK2, RBFOX1	13
synthesis of lipid	2.31E-09	AVPR1A, BDNF, estradiol, dopamine, EGR1, FOS, cortisol, MAPK10, MTMR2, noradrenalin, NR4A2, NR4A3, POMC, SNCA	6
metabolism of carbohydrate	2.41E-09	AVPR1A, BDNF, estradiol, DRD1, HLA-A, cortisol, IRS2, MTMR2, noradrenalin, NR4A2, NR4A3, POMC, SNCA	5
concentration of dopamine	2.57E-09	BDNF, estradiol, dopamine, MAPK10, NR4A2, POMC, SNCA	7
secretion of steroid hormone	2.86E-09	AVPR1A, BDNF, dopamine, EGR1, noradrenalin, POMC	10
stimulation of cells	3.14E-09	BDNF, estradiol, dopamine, DRD1, FOS, IgA, noradrenalin, NTRK2, POMC, RBFOX1, SNCA	9
quantity of dopaminergic	3.24E-09	estradiol, DRD1, NR4A2, NTRK2, SNCA	5

neurons			
motor function	3.70E-09	BDNF, estradiol, HLA-A, NR4A2, POMC, SNCA, SYT4	9
secretion of L-glutamic acid	3.78E-09	AVPR1A, BDNF, GRIN2B, NTRK2, SNCA	7
synthesis of DNA	3.90E-09	BDNF, estradiol, dopamine, DUSP1, FOS, cortisol, IRS2, noradrenalin, NR4A3, PHIP, POMC	11
degeneration of neurons	4.22E-09	BDNF, estradiol, dopamine, GRIN2B, MTMR2, NR4A2, NR4A3, NTRK2, SNCA	8
transport of molecule	4.46E-09	AVPR1A, BDNF, estradiol, dopamine, DOPEY2, DRD1, EGR1, GRIN2B, HLA-A, cortisol, IgA, IRS2, LASP1, noradrenalin, NR4A3, NTRK2, PHIP, POMC, SNCA, SYT4	9
release of amino acids	4.74E-09	BDNF, estradiol, dopamine, DRD1, NR4A2, POMC, SNCA	11
emotional behavior	4.81E-09	ARC, AVPR1A, BDNF, DRD1, GRIN2B, noradrenalin, NR4A2, NTRK2, POMC	11
release of neurotransmitter	5.73E-09	BDNF, estradiol, dopamine, cortisol, noradrenalin, NTRK2, SNCA, SYT4	12
long-term potentiation of cerebral cortex	5.75E-09	BDNF, estradiol, dopamine, DRD1, EGR1, NTRK2, SNCA	19
synthesis of carbohydrate	5.86E-09	AVPR1A, estradiol, HLA-A, cortisol, IRS2, MTMR2, noradrenalin, NR4A2, NR4A3, POMC, SNCA	7
place aversion	7.67E-09	ARC, DRD1, noradrenalin, POMC	12
secretion of molecule	7.73E-09	AVPR1A, BDNF, estradiol, dopamine, EGR1, GRIN2B, cortisol, noradrenalin, NTRK2, POMC, SNCA, SYT4	13
neuronal cell death	9.27E-09	BDNF, estradiol, dopamine, DUSP1, EGR1, FOS, GRIN2B, MAPK10, noradrenalin, NR4A2, NR4A3, NTRK2, SNCA	7
depressive disorder	9.42E-09	AVPR1A, BDNF, estradiol, dopamine, DRD1, GRIN2B, cortisol, noradrenalin, POMC	18
release of catecholamine	9.50E-09	BDNF, estradiol, dopamine, cortisol, noradrenalin, NTRK2, SNCA	8
organization of cytoplasm	9.87E-09	ARC, ARHGAP24, BDNF, estradiol, DOPEY2, DRD1, EGR1, FOS, GRIN2B, cortisol, LASP1, MAPK10, noradrenalin, NTRK2, PHIP, POMC, SNCA, SNX10	6
obesity	1.11E-08	AVPR1A, BDNF, estradiol, DUSP1, GRIN2B, IgA, IRS2, NR4A2, NR4A3, NTRK2, POMC, SYT4	8
secretion of steroid	1.13E-08	AVPR1A, BDNF, estradiol, dopamine, EGR1, noradrenalin, POMC	11
cell movement	1.14E-08	ARHGAP24, BDNF, estradiol, dopamine, DRD1, DUSP1, DUSP5, EGR1, FOS, FOSL2, HLA-A, cortisol, IgA, IRS2, LASP1, MAPK10, noradrenalin, NR4A2, NTRK2, POMC, SNCA, TET2	12
cell viability of cerebral cortex cells	1.42E-08	BDNF, estradiol, dopamine, EGR1, NTRK2, SNCA	14
coordination	1.47E-08	BDNF, dopamine, DRD1, GRIN2B, HLA-A, MAPK10, NR4A2, SYT4	17
necrosis of epithelial tissue	1.70E-08	BDNF, estradiol, dopamine, EGR1, FOS, GRIN2B, cortisol, noradrenalin, NR4A3, NTRK2, POMC, SNCA	8
quantity of steroid	1.73E-08	AVPR1A, BDNF, estradiol, DUSP1, EGR1, cortisol, IRS2, noradrenalin, NTRK2, POMC, SNCA	20
conditioning	1.75E-08	ARC, BDNF, dopamine, DRD1, GRIN2B, noradrenalin, POMC, SYT4	15
generation of cells	1.78E-08	ARHGAP24, AVPR1A, BDNF, estradiol, dopamine, DRD1, EGR1, FOS, FOSL2, FOXP2, HLA-A, cortisol, IRS2, MTMR2, NR4A2, NTRK2, POMC, SNCA, SNX10, TET2	6
organization of cytoskeleton	1.83E-08	ARC, ARHGAP24, BDNF, estradiol, DRD1, EGR1, FOS, GRIN2B, cortisol, LASP1, MAPK10, noradrenalin, NTRK2, PHIP, POMC, SNCA, SNX10	13
tauopathy	2.03E-08	ARC, BDNF, estradiol, dopamine, DRD1, GRIN2B, cortisol, MAPK10, noradrenalin, NR4A2, NTRK2, SNCA	7
neuromuscular disease	2.27E-08	BDNF, estradiol, dopamine, DRD1, DUSP5, EGR1, FOS, GRIN2B, cortisol, NR4A2, NTRK2, PKIA, RBFOX1, SNCA	10
cytotoxicity of cells	2.39E-08	estradiol, dopamine, FOS, GRIN2B, HLA-A, noradrenalin, POMC, SNCA	10
cell death of hippocampal neurons	2.75E-08	BDNF, estradiol, GRIN2B, MAPK10, NTRK2, SNCA	12
quantity of neurons	3.19E-08	BDNF, estradiol, DRD1, DUSP1, GRIN2B, MAPK10, NR4A2, NTRK2, POMC, SNCA	4
neurotransmission	3.30E-08	ARC, BDNF, estradiol, dopamine, DRD1, GRIN2B, HLA-A, noradrenalin,	15

		NTRK2, SNCA	
Movement Disorders	3.40E-08	BDNF, dopamine, DRD1, DUSP5, EGR1, FOS, GRIN2B, cortisol, MTMR2, noradrenalin, NR4A2, NTRK2, PKIA, RBFOX1, SNCA	11
apoptosis of brain cells	3.78E-08	BDNF, estradiol, dopamine, EGR1, FOS, MAPK10, NTRK2	4
disorder of basal ganglia	4.02E-08	BDNF, dopamine, DRD1, DUSP5, EGR1, FOS, GRIN2B, cortisol, NR4A2, NTRK2, PKIA, RBFOX1, SNCA	5
release of L-glutamic acid	4.04E-08	BDNF, estradiol, dopamine, DRD1, NR4A2, SNCA	9
Mood Disorders	4.35E-08	AVPR1A, BDNF, estradiol, dopamine, DRD1, GRIN2B, cortisol, noradrenalin, NTRK2, POMC	11
activation of cerebral cortex cells	4.55E-08	BDNF, estradiol, dopamine, noradrenalin	6
neurological signs	4.66E-08	BDNF, dopamine, DRD1, DUSP5, EGR1, FOS, GRIN2B, cortisol, NTRK2, PKIA, RBFOX1, SNCA	16
progressive motor neuropathy	4.67E-08	BDNF, estradiol, dopamine, DRD1, EGR1, GRIN2B, cortisol, NR4A2, NTRK2, RBFOX1, SNCA	5
apoptosis of granule cells	6.16E-08	BDNF, dopamine, EGR1, FOS, NTRK2	6
synaptic transmission	6.69E-08	ARC, BDNF, estradiol, dopamine, DRD1, GRIN2B, HLA-A, noradrenalin, SNCA	11
binding of DNA	6.90E-08	estradiol, dopamine, EGR1, FOS, FOSL2, FOXP2, cortisol, noradrenalin, NR4A2, POMC, SNCA	11
activation of neurons	7.04E-08	BDNF, estradiol, dopamine, FOS, noradrenalin, SNCA	6
hyperphagia	7.04E-08	BDNF, DRD1, GRIN2B, IRS2, NTRK2, POMC	6
quantity of metal	7.45E-08	AVPR1A, BDNF, estradiol, dopamine, DRD1, FOS, GRIN2B, HLA-A, cortisol, noradrenalin, POMC	11
locomotion	8.04E-08	BDNF, estradiol, dopamine, DRD1, DUSP1, MAPK10, NR4A2, NTRK2, SNCA	9
spatial memory	8.92E-08	AVPR1A, BDNF, estradiol, dopamine, noradrenalin, POMC	6
neurodegeneration of cerebral cortex	9.26E-08	BDNF, estradiol, GRIN2B, NTRK2, SNCA	5
Huntington's Disease	9.33E-08	BDNF, dopamine, DRD1, DUSP5, EGR1, FOS, GRIN2B, cortisol, NTRK2, PKIA, RBFOX1	11

Table S5: Functional enrichment of the top 29 musical ability-related molecules. Note that the significance of these best-enriched functions is notably weaker than in the other functional enrichment results with similar number of molecules (Table S6 and S7). The musical ability-related genes may for example have diverse functions or their function is unknown. The functions with less than three members should be considered with caution.

Diseases or functions annotation	p-value	Molecules	# molecules
cell death of granule cells	1.1E-06	CASP6, EGR1, FOS, GRID2	4
development of head	2.0E-06	ATOH1, BMPR1B, CASP6, EGF, EGR1, FOS, FOXP2, SEC24B, UNC5C	9
development of bone marrow cells	7.8E-06	AVPR1A, EGF, EGR1, FOS	4
development of cochlear nucleus	8.5E-06	ATOH1, SEC24B	2
apoptosis of neurons	1.0E-05	ATOH1, BMPR1B, CASP6, EGR1, FOS, GRID2	6
neuronal cell death	1.4E-05	ATOH1, BMPR1B, CASP6, EGF, EGR1, FOS, GRID2	7
cell death of brain	1.8E-05	CASP6, EGF, EGR1, FOS, GRID2	5
contact growth inhibition	2.3E-05	BMPR1B, CASP6, EGF, EGR1	4
baroreceptor reflex	2.4E-05	AVPR1A, FOS	2
formation of brain cells	3.1E-05	ATOH1, FOXP2, UNC5C	3
invasion of fibroblast cell lines	3.7E-05	EGF, GRID2, UNC5C	3
differentiation of cerebellar granule cell	3.8E-05	ATOH1, GRID2	2
colony formation of carcinoma cell lines	3.8E-05	EGF, EGR1, UNC5C	3
synthesis of GABA	4.7E-05	EGF, EGR1	2
development of neurons	5.2E-05	ATOH1, CASP6, EGF, EGR1, FOS, GRID2, UNC5C	7
development of sensory organ	5.4E-05	ATOH1, BMPR1B, CASP6, EGF, EGR1, FOS	6
proliferation of chondrocytes	6.4E-05	BMPR1B, EGF, FOS	3
development of neutrophils	6.6E-05	EGR1, FOS	2
differentiation of tumor cells	8.7E-05	EGF, EGR1, FOS	3
synthesis of dinoprost	8.9E-05	EGF, HPGDS	2
contact growth inhibition of tumor cell lines	9.2E-05	BMPR1B, EGF, EGR1	3
abnormal morphology of head	9.9E-05	ATOH1, BMPR1B, CASP6, EGF, EGR1, FOS, GRID2	7
dissociation of cells	1.0E-04	EGF, GRID2	2
development of connective tissue	1.1E-04	AVPR1A, BMPR1B, EGF, EGR1, FOS	5
development of central nervous system	1.1E-04	ATOH1, EGF, EGR1, FOXP2, SEC24B, UNC5C	6
learning	1.4E-04	AVPR1A, BMPR1B, EGR1, FOS, GRID2	5
invasion of embryonic cell lines	1.4E-04	EGF, FOS	2
transformation of fibroblast cell lines	1.8E-04	EGF, EGR1, FOS, GRID2	4
formation of eye	1.8E-04	BMPR1B, CASP6, EGF, EGR1, FOS	5
secretion of steroid	1.9E-04	AVPR1A, EGF, EGR1	3

Table S6: Functional enrichment of the top 18 music listening-related molecules.

Diseases or Functions Annotation	p-Value	Molecules	# Molecules
learning	2.6E-12	ARC, BDNF, beta-estradiol, CREB1, dopamine, EGR1, FOS, hydrocortisone, norepinephrine, NR4A2	10
synthesis of lipid	3.9E-11	AKR1C3, BDNF, beta-estradiol, CREB1, dopamine, EGR1, FOS, hydrocortisone, norepinephrine, NR4A2, NR4A3	11
proliferation of muscle cells	5.1E-11	BDNF, beta-estradiol, CREB1, EGR1, FOS, norepinephrine, NR4A2, NR4A3, TNFRSF10B	9
short-term memory	7.4E-11	BDNF, beta-estradiol, CREB1, dopamine, hydrocortisone	5
memory	8.5E-11	ARC, BDNF, beta-estradiol, CREB1, dopamine, EGR1, hydrocortisone, norepinephrine	8
synthesis of steroid	1.0E-10	AKR1C3, BDNF, beta-estradiol, CREB1, dopamine, hydrocortisone, NR4A2, NR4A3	8
proliferation of smooth muscle cells	1.5E-10	beta-estradiol, CREB1, EGR1, FOS, norepinephrine, NR4A2, NR4A3, TNFRSF10B	8
cellular homeostasis	5.1E-10	BDNF, beta-estradiol, CREB1, dopamine, EGR1, FOS, GABARAPL2, HLA-A, hydrocortisone, Iga, MYADM, norepinephrine, NR4A2, TNFRSF10B	14
behavior	1.0E-09	ARC, BDNF, beta-estradiol, CREB1, dopamine, EGR1, FOS, hydrocortisone, norepinephrine, NR4A2, NR4A3	11
activation of blood cells	1.1E-09	BDNF, beta-estradiol, dopamine, FOS, GP9, HLA-A, Iga, norepinephrine, NR4A2, NR4A3, TNFRSF10B	11
activation of cerebral cortex cells	1.3E-09	BDNF, beta-estradiol, dopamine, norepinephrine	4
long-term memory	1.8E-09	ARC, BDNF, CREB1, dopamine, EGR1	5
growth of tumor	2.4E-09	AKR1C3, BDNF, beta-estradiol, CREB1, dopamine, EGR1, FOS, norepinephrine, NR4A2, TNFRSF10B	10
necrosis of epithelial tissue	3.7E-09	BDNF, beta-estradiol, dopamine, EGR1, FOS, hydrocortisone, norepinephrine, NR4A3, TNFRSF10B	9
quantity of catecholamine	3.9E-09	BDNF, beta-estradiol, dopamine, hydrocortisone, norepinephrine, NR4A2	6
long-term potentiation	3.9E-09	ARC, BDNF, beta-estradiol, CREB1, dopamine, EGR1, norepinephrine	7
synthesis of DNA	4.9E-09	BDNF, beta-estradiol, CREB1, dopamine, FOS, hydrocortisone, norepinephrine, NR4A3	8
apoptosis of neurons	5.0E-09	BDNF, beta-estradiol, CREB1, dopamine, EGR1, FOS, NR4A2, NR4A3	8
maturation of cells	6.4E-09	BDNF, beta-estradiol, CREB1, EGR1, FOS, hydrocortisone, Iga, norepinephrine, NR4A2	9
activation of leukocytes	1.4E-08	BDNF, beta-estradiol, dopamine, FOS, HLA-A, Iga, norepinephrine, NR4A2, NR4A3, TNFRSF10B	10
neuronal cell death	1.5E-08	BDNF, beta-estradiol, CREB1, dopamine, EGR1, FOS, norepinephrine, NR4A2, NR4A3	9
transactivation of RNA	2.6E-08	BDNF, beta-estradiol, CREB1, EGR1, FOS, hydrocortisone, NR4A2, NR4A3	8
activation of neurons	2.8E-08	BDNF, beta-estradiol, dopamine, FOS, norepinephrine	5
inhibition of pyramidal neurons	3.0E-08	BDNF, dopamine, norepinephrine	3
metabolism of hormone	3.2E-08	AKR1C3, beta-estradiol, CREB1, dopamine, hydrocortisone, norepinephrine	6
spatial memory	3.4E-08	BDNF, beta-estradiol, CREB1, dopamine, norepinephrine	5
binding of DNA	4.4E-08	beta-estradiol, CREB1, dopamine, EGR1, FOS, hydrocortisone, norepinephrine, NR4A2	8
diabetes mellitus	4.7E-08	AKR1C3, BDNF, beta-estradiol, EGR1, FOS, HLA-A, hydrocortisone, norepinephrine, NR4A2, NR4A3	10
motor function	4.8E-08	BDNF, beta-estradiol, CREB1, HLA-A, NR4A2	5
development of lymphocytes	6.1E-08	BDNF, beta-estradiol, CREB1, dopamine, EGR1, FOS, HLA-A, NR4A2	8

Table S7: Functional enrichment of the top 29 music practice-related molecules. The functions with less than three members should be considered with caution.

Diseases or functions annotation	p-value	Molecules	# molecules
epileptic seizure	7.7E-11	ARC, BDNF, DNAJB5, DUSP1, DUSP5, EGR1, FOS, MAPK10	8
transformation of fibroblast cell lines	4.2E-09	EGF, EGR1, EIF4E, FBXO7, FOS, MAPK10, MXI1, ODC1	8
expression of RNA	2.8E-08	BDNF, BLOC1S2, DNAJB5, dopamine, DRD1, DUSP1, DUSP5, EGF, EGR1, EIF4E, FOS, FOXP2, MAPK10, MGEA5, MXI1, ODC1, SNCA	17
cell death of brain	4.1E-08	BDNF, dopamine, DUSP1, EGF, EGR1, FOS, MAPK10, SNCA	8
cognition	8.3E-08	ARC, BDNF, dopamine, DOPEY2, DRD1, EGR1, FOS, MAPK10, SNCA	9
excitation of neurons	1.6E-07	BDNF, dopamine, DRD1, FOS, SNCA	5
transcription of RNA	1.6E-07	BDNF, BLOC1S2, DNAJB5, dopamine, DRD1, DUSP1, DUSP5, EGF, EGR1, FOS, FOXP2, MAPK10, MGEA5, MXI1, SNCA	15
differentiation of tumor cell lines	2.0E-07	BDNF, DUSP1, EGF, EGR1, FOS, ODC1, SNCA, UBE2D3	8
apoptosis of brain	2.1E-07	BDNF, dopamine, DUSP1, EGR1, FOS, MAPK10	6
apoptosis of dopaminergic neurons	2.3E-07	dopamine, MAPK10, SNCA	3
cell death of dopaminergic neurons	4.2E-07	BDNF, dopamine, MAPK10, SNCA	4
cell death of brain cells	5.1E-07	BDNF, dopamine, DUSP1, EGR1, FOS, MAPK10, SNCA	7
concentration of dopamine	6.6E-07	BDNF, dopamine, EGF, MAPK10, SNCA	5
uptake of neurotransmitter	8.4E-07	BDNF, EGF, FOS, SNCA	4
apoptosis of granule cells	1.0E-06	BDNF, dopamine, EGR1, FOS	4
long-term potentiation of cerebral cortex	1.2E-06	BDNF, dopamine, DRD1, EGR1, SNCA	5
cell death of striatal neurons	1.2E-06	BDNF, dopamine, DUSP1, EGF	4
apoptosis of tumor cell lines	1.6E-06	BDNF, dopamine, DUSP1, EGF, EGR1, EIF4E, FOS, MAPK10, MGEA5, MXI1, ODC1, SNCA	12
activation of striatal neurons	1.6E-06	BDNF, dopamine	2
synaptic transmission of cerebral cortex cells	1.6E-06	BDNF, dopamine, DRD1, SNCA	4
locomotion	1.7E-06	BDNF, dopamine, DRD1, DUSP1, EGF, MAPK10, SNCA	7
fear	1.7E-06	ARC, BDNF, DRD1, EIF4E	4
invasion of embryonic cell lines	1.9E-06	EGF, FOS, ODC1	3
long-term memory	2.2E-06	ARC, BDNF, dopamine, EGR1	4
localization of lysosome	2.2E-06	BLOC1S2, EGF, SNCA	3
apoptosis of striatal neurons	2.5E-06	BDNF, dopamine, DUSP1	3
cell death of tumor cell lines	2.8E-06	BDNF, dopamine, DUSP1, EGF, EGR1, EIF4E, FOS, MAPK10, MGEA5, MXI1, ODC1, SNCA, WAPL	13
cell viability of neurons	3.0E-06	BDNF, dopamine, EGF, EGR1, MAPK10, SNCA	6
abnormality of striatum	3.0E-06	BDNF, dopamine, DRD1, SNCA	4
development of striatum	4.4E-06	BDNF, DRD1, FOXP2	3

Table S8: Localization information of the top genes

Gene symbol	Main function	Main cellular location	Localization of music-related information
EGR1	Transcription factor	Nucleus	Multiple regions of the brain: songbirds e.g. NCM, area X, LMAN, RA; Pallium in frogs. Differences between producing and listening
Cortisol	Hormone	Extracellular space	Blood, saliva
FOS	Transcription factor	Nucleus, cytosol, endoplastic reticulum	Multiple regions of the brain: songbirds e.g. NCM, HVC, RA. Differences between producing and listening
FOXP2	Transcription factor	Nucleus	
ARC	Synaptic plasticity, cell morphology and cytoskeletal organization	Plasma membrane	Multiple regions of the brain: songbirds e.g. NCM, CMM, HVC, RA. Differences between producing and listening
Dopamine	Neurotransmitter, hormone, chemical messenger	Extracellular space	Dorsal and ventral striatum in humans; Neostriatum in rats; Area X in zebra finch
BDNF	Neurotrophin receptor	Extracellular space	Chicken hippocampus; Mouse hypothalamus and auditory cortex; Canary HVC; Zebra finch HVC, LMAN, RA, area X
Noradrenalin	Hormone, neurotransmitter	Extracellular space	Human and chicken blood
GRIN2B	NMDA receptor	Plasma membrane	Rat auditory cortex; Zebra finch multiple regions including LMAN and area X
SYT4	Ca(2+)-dependent trafficking, dendrite formation	Plasma membrane	Zebra finch multiple regions including AL, LMAN, HVC, RA
PHIP	Insulin growth factor signaling, cell morphology and cytoskeletal organization	Extracellular space, nucleus	Zebra finch multiple regions including NCM, AL, area X
MAPK10	Neuronal proliferation, differentiation, migration and programmed cell death		Zebra finch area X, NCM, HVC
DRD1	Dopamine receptor	Plasma membrane, cytosol	Starling POM; Zebra finch area X
SNCA	Neuronal responsiveness, dopamine regulation	Ubiquitous	NA
NR4A3	Transcription factor, nuclear hormone receptor	Nucleus	Zebra finch multiple regions including area X, AL, NCM
IRS2	Insulin receptor substrate	Cytosol, nucleus, plasma membrane	
ARHGAP24	Rho GTPase-activator	Cytoskeleton, cytosol	Human blood; Zebra finch RA
MTMR2	Phosphatase	Extracellular space, cytosol, nucleus	Zebra finch area X, AL, LMAN
NR4A2	Transcription factor, nuclear hormone receptor	Nucleus	Zebra finch area X, AL; Mouse forebrain cortex
DUSP1	Phosphatase	Nucleus, cytosol	Zebra finch multiple regions including area X, NCM, LMAN
DUSP5	Phosphatase	Nucleus	Zebra finch multiple regions including area X, AL, NCM
PKIA	cAMP dependent protein kinase inhibitor	Nucleus	Zebra finch area X, AL
PNISR	Splicing factor	Nucleus, cytosol	Zebra finch multiple regions including area X, NCM, AL
Estradiol	Hormone	Extracellular space	Human saliva; Sparrows and zebra finch NCM;

			Canary HVC
TET2	Dioxygenase, DNA demethylation	Nucleus	Zebra finch: area X, AL
UBE2D3	Ubiquitin-conjugating enzyme	Extracellular space, nucleus, cytosol	Zebra finch area X
FAM13A	GTPase activator	Cytosol	Zebra finch area X
NUDT9	ADP-ribose pyrophosphatase	Extracellular space, mitochondrion	Zebra finch area X, HVC
DOPEY2	May be involved in protein traffic	Golgi apparatus, extracellular space	Zebra finch area X, LMAN
NTRK2	Neurotrophin receptor kinase	Plasma membrane, cytosol, endosome	Mouse auditory cortex; Zebra finch area X, AL

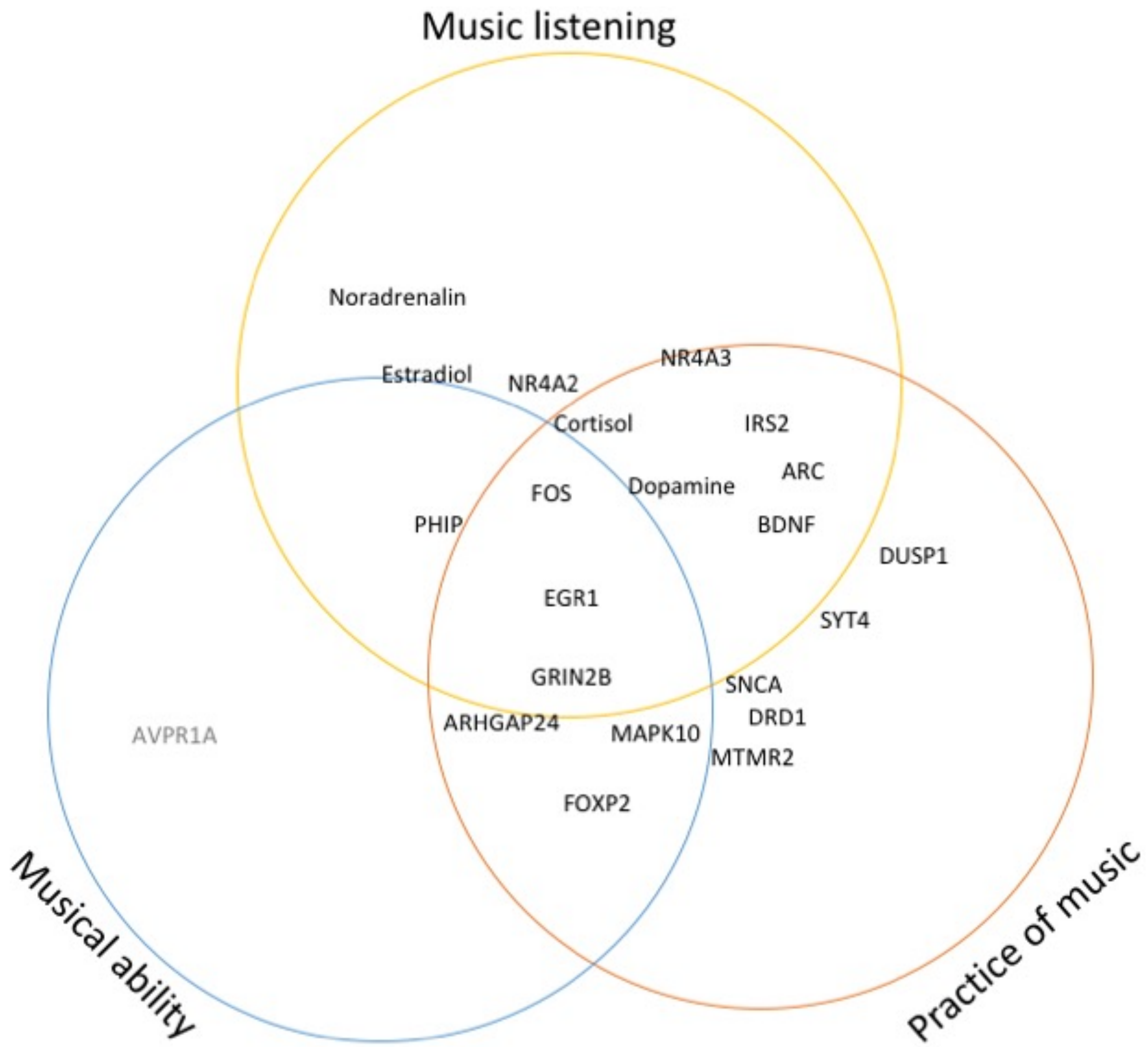

Figure S1. Evidence for the top 20 genes from different subphenotypes. The genes showing evidence in the subphenotypes are shown within the circles, or when there is only single evidence, on the border of the circle. Almost half of the genes show evidence with both practice of music and music listening as shown in the intersection. The *AVPR1A* was among top 3 of the CE analysis of the musical ability-related studies, but not among top 20 in the analysis of the complete data.

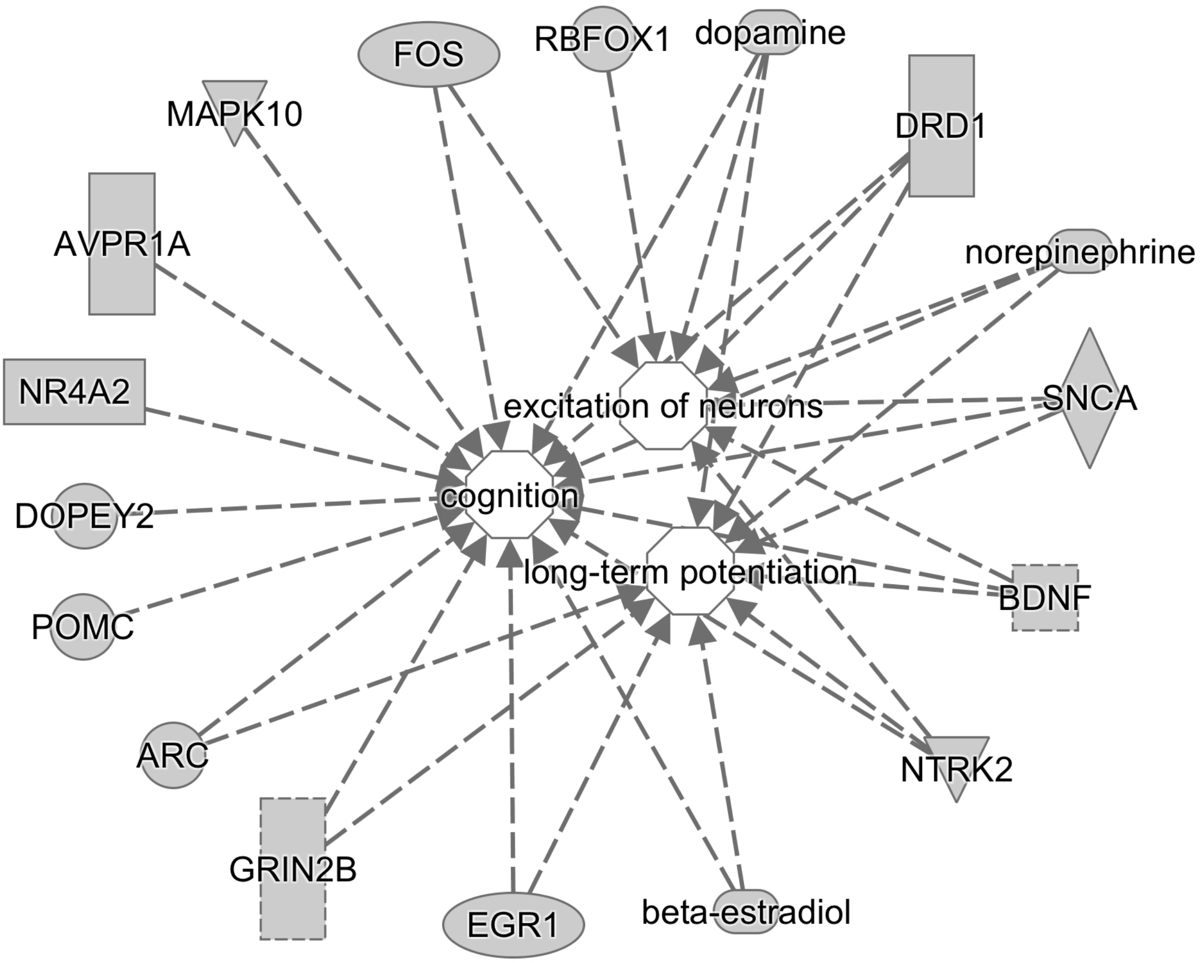

Figure S2. Enrichment analysis of the top 40 genes showed cognition, excitation of neurons and long-term potentiation. Genes and molecules associated to these functions are shown by nodes connected with arrows to the function. These three functions, that were found to be enriched among the top 40 genes and molecules, are associated partially to the same top ranked genes and molecules as shown in this figure. The illustration was generated through the use of IPA.

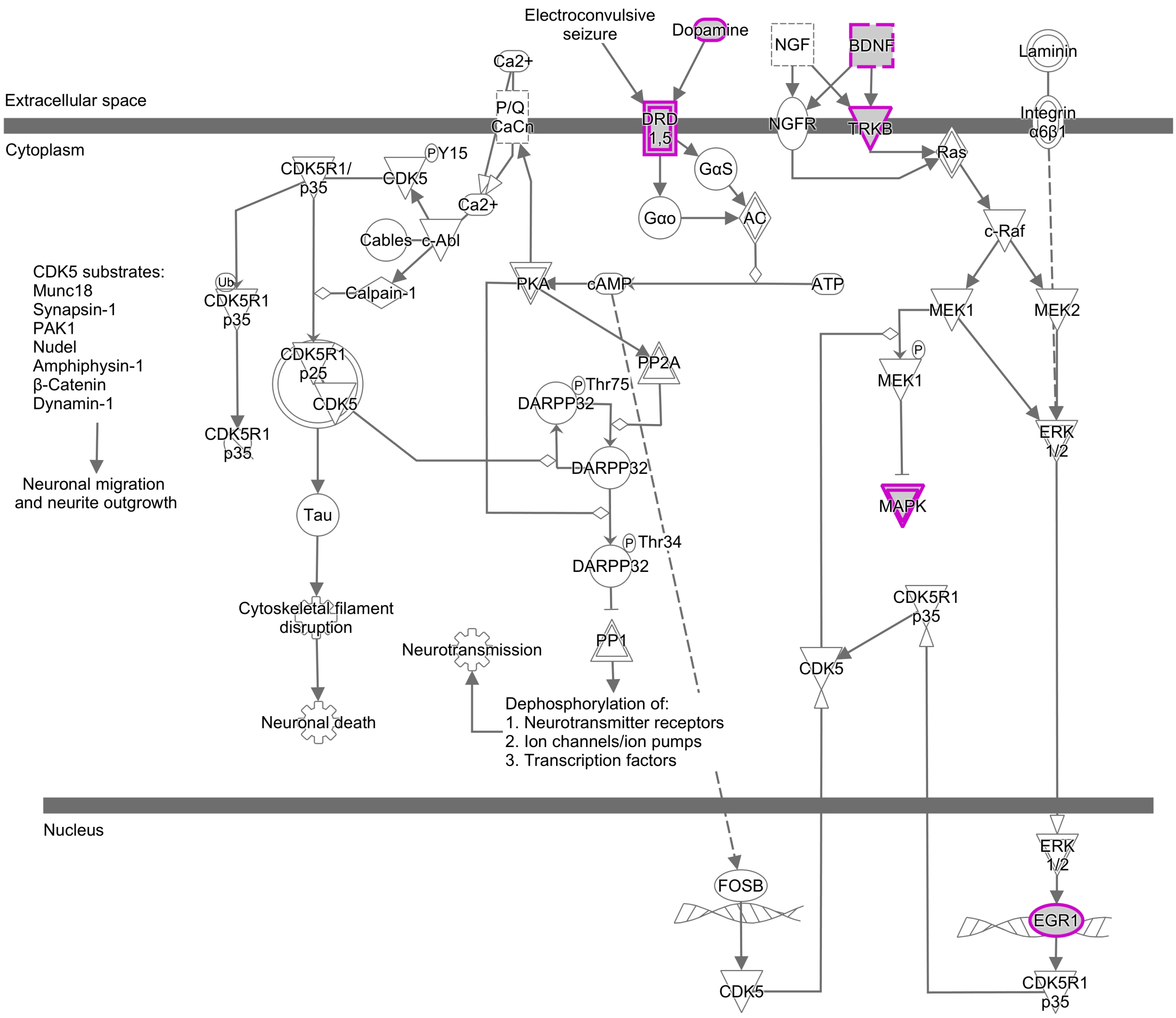

Figure S3. CDK5 signaling pathway was enriched among the top 40 prioritized molecules. The 6 molecules that were among the top 40 molecules are coloured as pink. Additionally, MEK1/2 (*MAP2K1* and *MAP2K2*) has been suggested to be important for song learning[45]. Overall, the pathway is important in neuronal development and cognition, including learning and memory[44]. As expected from the pathway, *MAPK10* has been shown to be downregulated[39] and *EGR1*, dopamine and *NTRK2* (TRKB in the figure) upregulated in music[S11, S77]. Notably, the pathway includes the *SYN1* gene, which is not found in birds[S99]. The illustration was generated through the use of IPA.

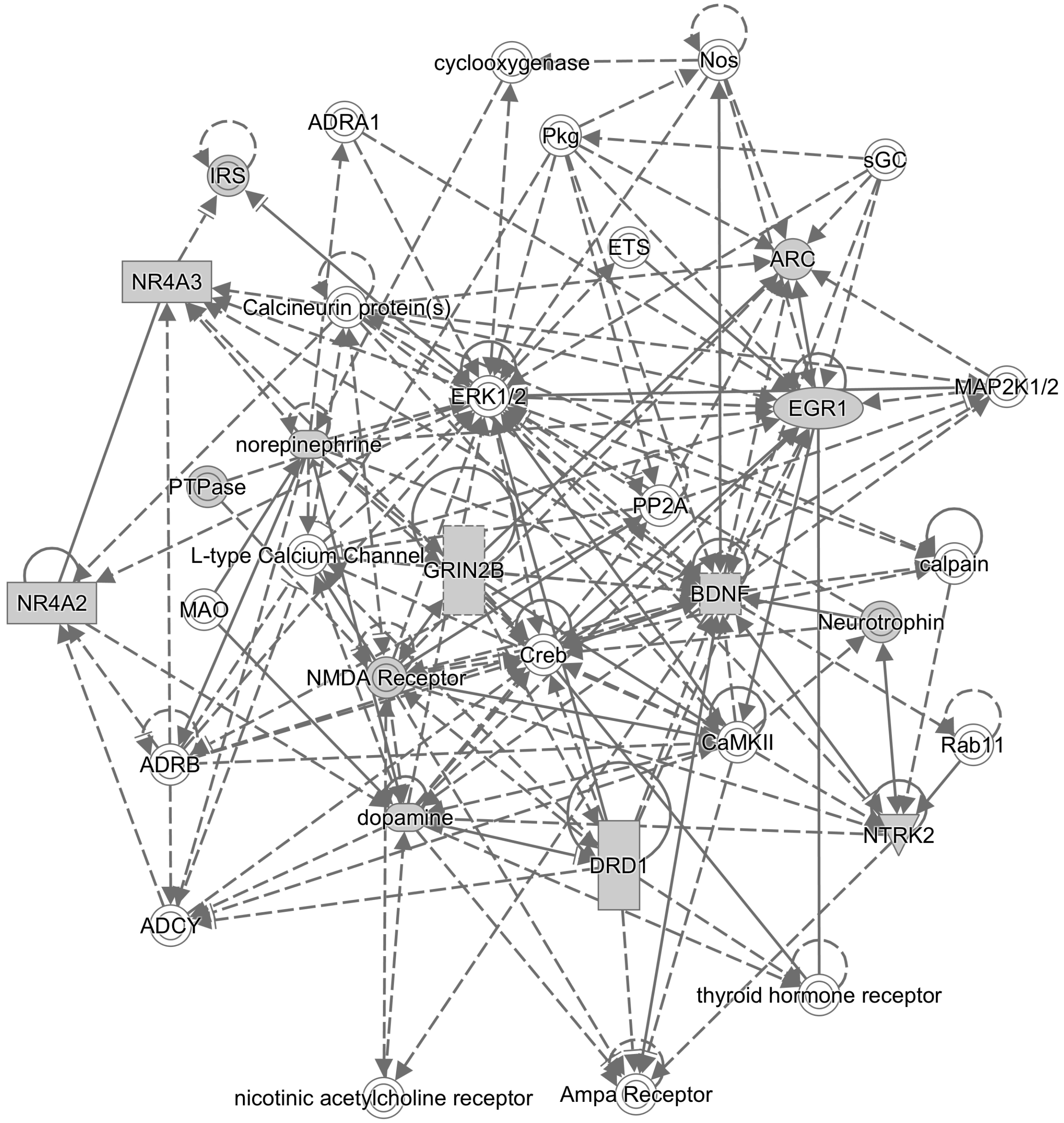

Figure S4. Behaviour-related interaction network including ten of the top 40 genes. The genes are marked as nodes. The grey nodes denote the ones included in the top 40 list. The most enriched function among all the genes within this interaction network was behaviour. All of the ten top genes within this network were also associated with behaviour. Other enriched functions were nervous system development and function, and cell-to-cell signaling and interaction. The analysis and illustration was generated through the use of IPA where the network got score of 21.

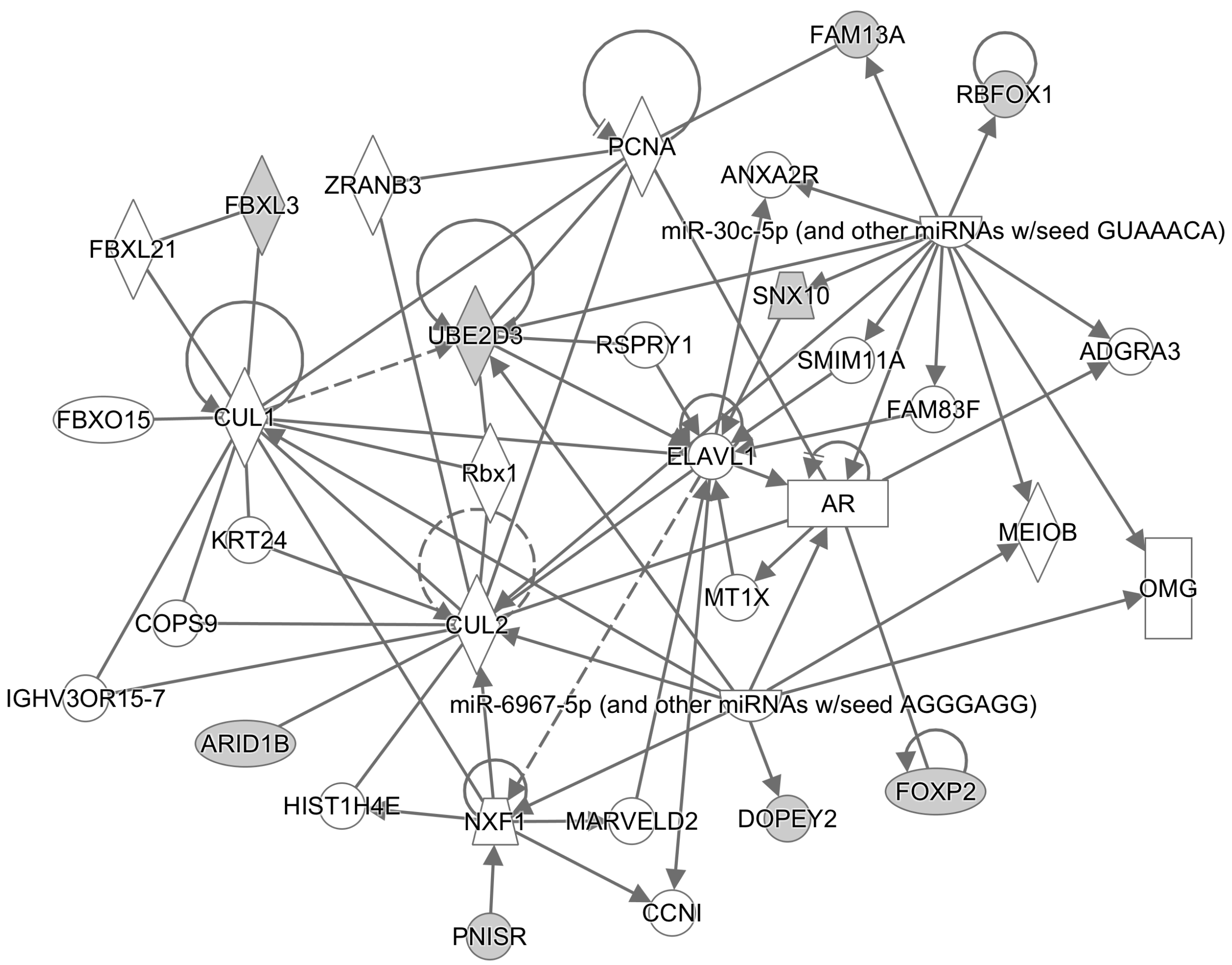

Figure S5. Interaction network including nine of the top 40 genes. The genes are marked as nodes. Grey nodes denote the ones included in the top 40 list. The most enriched functions among all the genes in this network included post-translational modification, cell cycle and DNA replication, recombination and repair. The analysis and illustration was generated through the use of IPA where the network got score of 19.

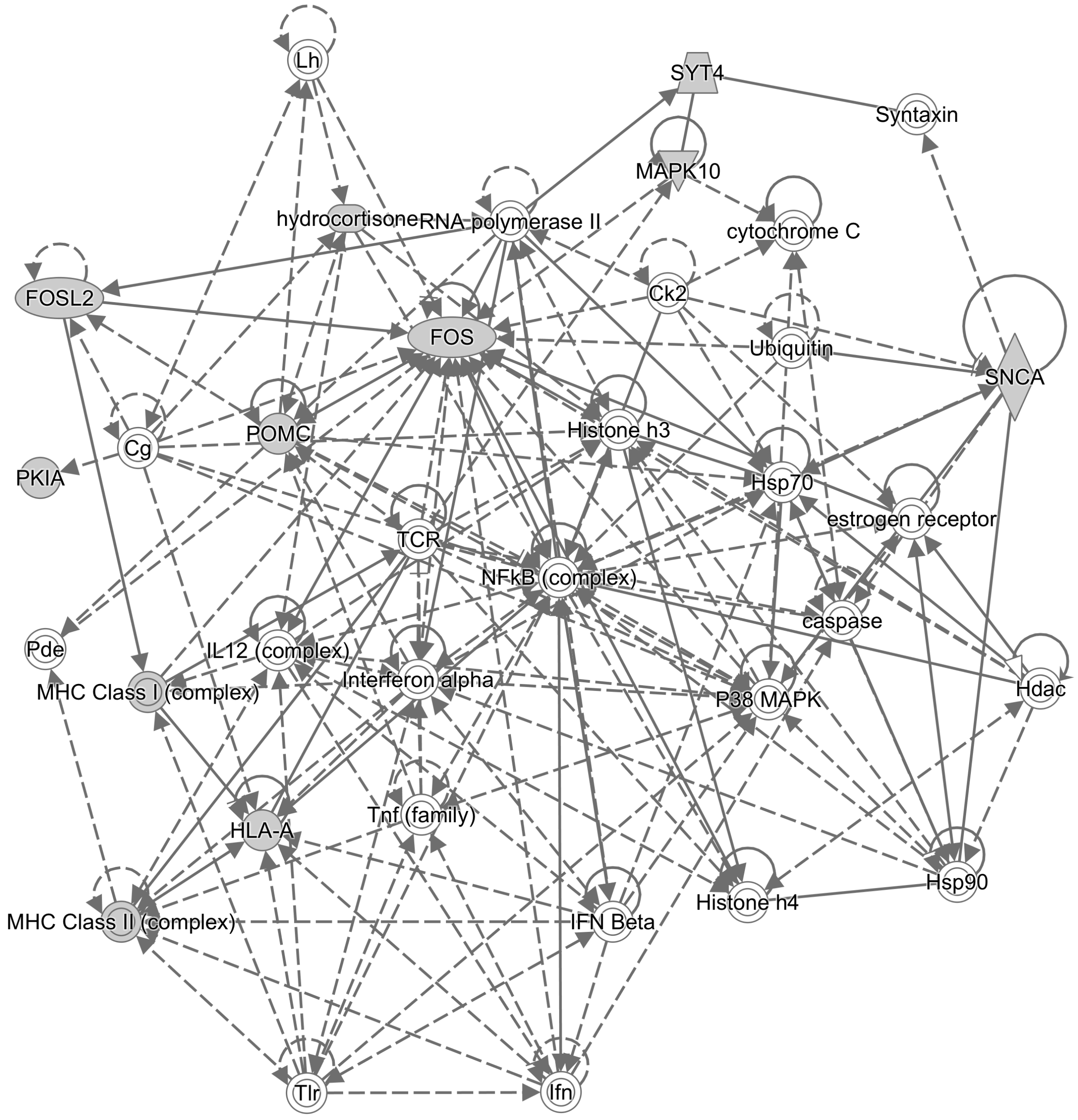

Figure S6. Interaction network including nine of the top 40 genes. The genes are marked as nodes and grey nodes denote the ones included in the top 40 list. The top functions associated with all the genes within this network included nervous system development, skeletal and muscular system development and cell-to-cell signaling. The analysis and illustration was generated through the use of IPA where the network got score of 19.

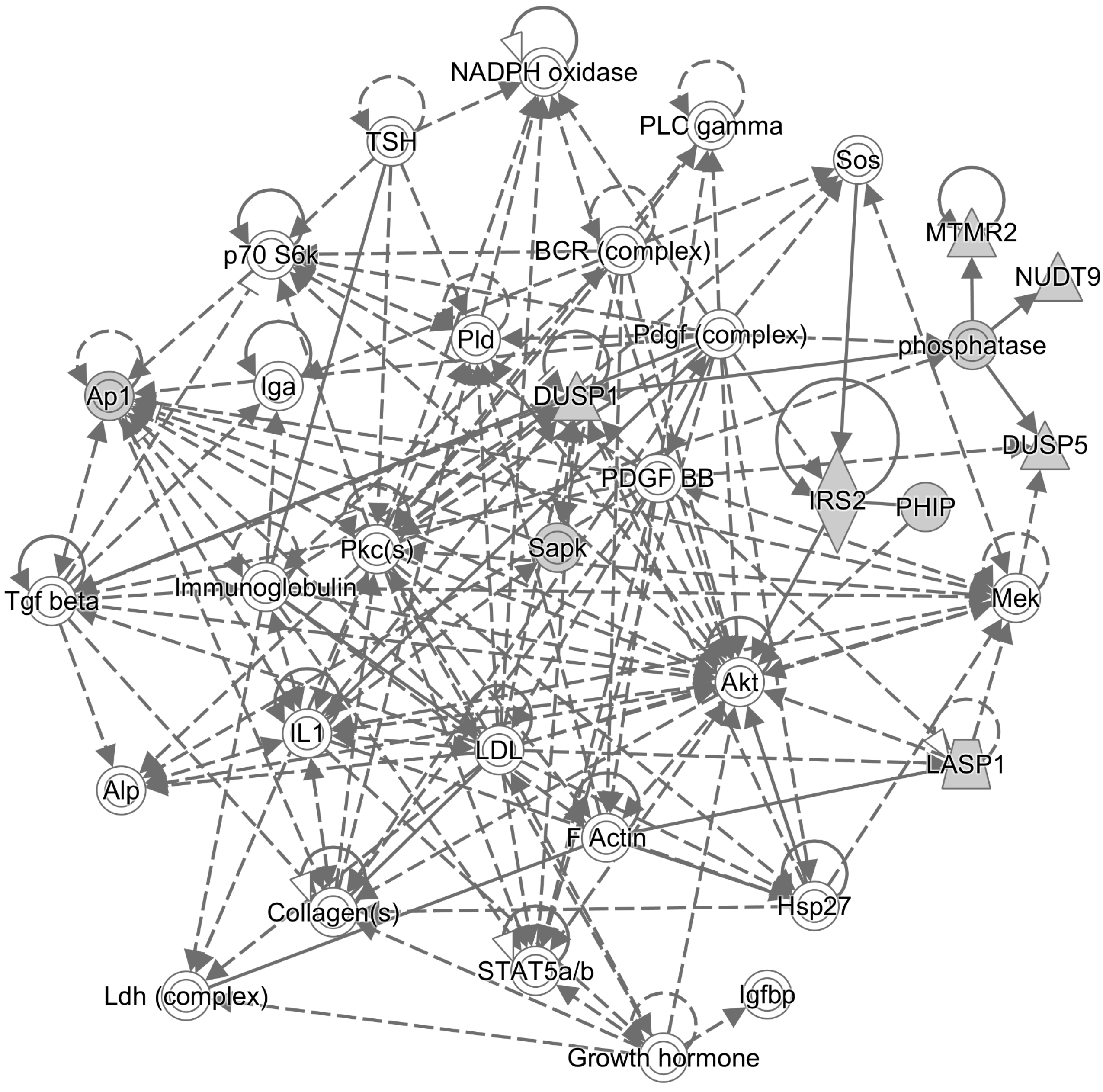

Figure S7. Interaction network including eight of the top 40 genes. The genes are marked as nodes and grey nodes denote the ones included in the top 40 list. Functions related to the genes within this network included post-translational modification, DNA replication and nucleic acid metabolism. The analysis and illustration was generated through the use of IPA where the network got score of 16.

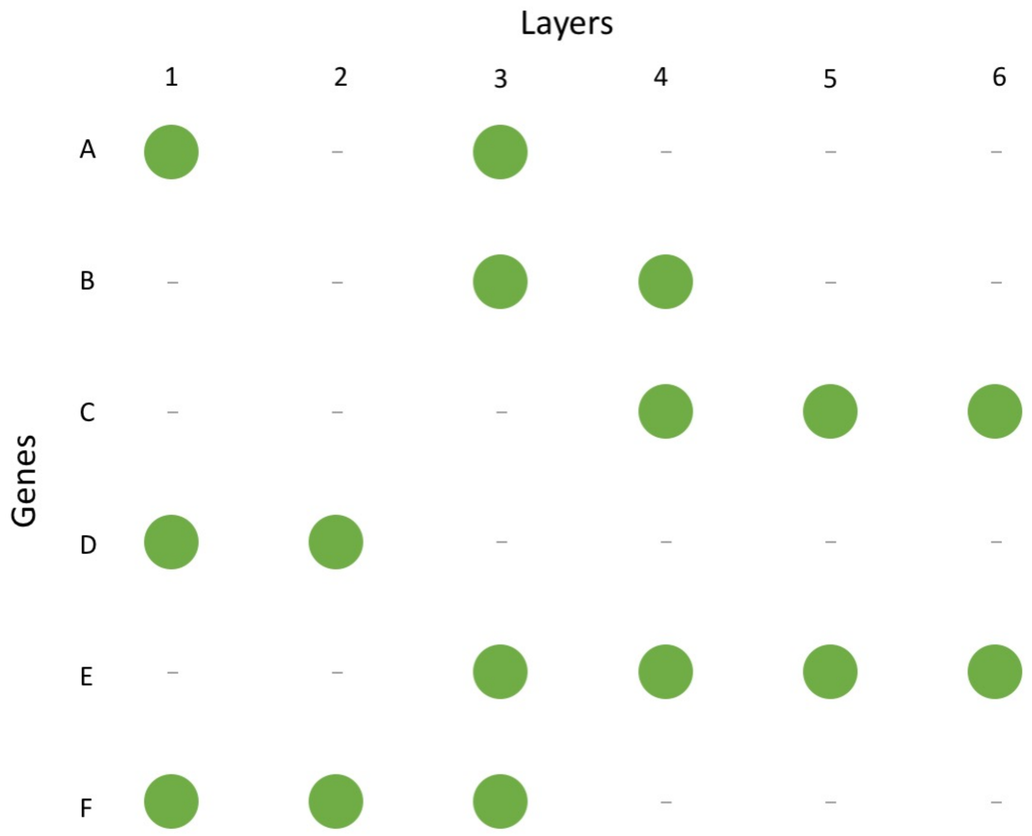

Figure S8. Calculation of convergent evidence (CE) scores. This illustration shows six evidence layers. The green dots represent the detection of a gene, while the dashes indicate the absence of evidence or negative result within each evidence layer. Each evidence layer is assigned a custom weight depending upon importance. For instance, let us assume that the custom weights of layers (1-6) are 1.0,1.0,0.9,0.8,0.6 and 0.5, respectively. Here, genes A and B are detected twice each. However, based on a weighted vote counting method, the convergent evidence scores of genes A and B would be 1.9 and 1.7 respectively.

Supplementary Information

List of studies included in the convergent analysis

S1. Abe, K., Matsui, S. & Watanabe, D. Transgenic songbirds with suppressed or enhanced activity of CREB transcription factor. *P. Natl. Acad. Sci. U.S.A.* **112**, 7599-7604 (2015).

S2. Angelucci, F., Ricci, E., Padua, L., Sabino, A. & Tonali, P.A. Music exposure differentially alters the levels of brain-derived neurotrophic factor and nerve growth factor in the mouse hypothalamus. *Neurosci. Lett.* **429**, 152-155 (2007).

S3. Arnauld, E., Jeantet, Y., Arsaut, J. & Demotes-Mainard, J. Involvement of the caudal striatum in auditory processing: c-fos response to cortical application of picrotoxin and to auditory stimulation. *Brain Res. Mol. Brain Res.* **41**, 27-35 (1996).

S4. Avey, M.T., Kanyo, R.A., Irwin, E.L. & Sturdy, C.B. Differential effects of vocalization type, singer and listener on ZENK immediate early gene response in black-capped chickadees (Poecile atricapillus). *Behav. Brain. Res.* **188**, 201-208 (2008).

S5. Bailey, D.J. & Wade, J. Differential expression of the immediate early genes FOS and ZENK following auditory stimulation in the juvenile male and female zebra finch. *Brain Res. Mol. Brain Res.* **116**, 147-154 (2003).

S6. Bartlett, D., Kaufman, D. & Smeltekop, R. The Effects of Music Listening and Perceived Sensory Experiences on the Immune-System as Measured by Interleukin-1 and Cortisol. *J. Music Ther.* **30**, 194-209 (1993).

S7. Basham, M.E., Nordeen, E.J. & Nordeen, K.W. Blockade of NMDA receptors in the anterior forebrain impairs sensory acquisition in the zebra finch (Poephila guttata). *Neurobiol. Learn. Mem.* **66**, 295-304 (1996).

S8. Bolhuis, J.J., Zijlstra, G.G.O., den Boer-Visser, A.M. & Van der Zee, E.A. Localized neuronal activation in the zebra finch brain is related to the strength of song learning. *P. Natl. Acad. Sci. U.S.A.* **97**, 2282-2285 (2000).

S9. Chaudhury, S. & Wadhwa, S. Prenatal auditory stimulation alters the levels of CREB mRNA, p-CREB and BDNF expression in chick hippocampus. *Int. J. Dev. Neurosci.* **27**, 583-590 (2009).

S10. Chen, Q., Heston, J.B., Burkett, Z.D. & White, S.A. Expression analysis of the speech-related genes FoxP1 and FoxP2 and their relation to singing behavior in two songbird species. *J. Exp. Biol.* **216**, 3682-3692 (2013).

S11. Chikahisa, S. *et al.* Exposure to music in the perinatal period enhances learning performance and alters BDNF/TrkB signaling in mice as adults. *Behav. Brain. Res.* **169**, 312-319 (2006).

S12. DeVries, M.S., Cordes, M.A., Stevenson, S.A. & Riters, L.V. Differential relationships between D1 and D2 dopamine receptor expression in the medial preoptic nucleus and sexually-motivated song in male European starlings (Sturnus vulgaris). *Neuroscience* **301**, 289-297 (2015).

S13. Dong, S. & Clayton, D.F. Partial dissociation of molecular and behavioral measures of song habituation in adult zebra finches. *Genes Brain Behav.* **7**, 802-809 (2008).

S14. Drnevich, J. *et al.* Impact of experience-dependent and -independent factors on gene expression in songbird brain. *P. Natl. Acad. Sci. U.S.A.* **109 Suppl 2**, 17245-17252 (2012).

S15. Eda-Fujiwara, H., Satoh, R., Bolhuis, J.J. & Kimura, T. Neuronal activation in female budgerigars is localized and related to male song complexity. *Eur. J. Neurosci.* **17**, 149-154 (2003).

S16. Emanuele, E. *et al.* Increased dopamine DRD4 receptor mRNA expression in lymphocytes of musicians and autistic individuals: bridging the music-autism connection. *Neuro Endocrinol. Lett.* **31**, 122-125 (2010).

S17. Fancourt, D., Aufegger, L. & Williamon, A. Low-stress and high-stress singing have contrasting effects on glucocorticoid response. *Front. Psychol.* **6**, 1242 (2015).

S18. Fancourt, D. & Williamon, A. Attending a concert reduces glucocorticoids, progesterone

and the cortisol/DHEA ratio. *Public Health* **132**, 101-104 (2016).
S19. Fukui, H. & Toyoshima, K. Influence of music on steroid hormones and the relationship between receptor polymorphisms and musical ability: a pilot study. *Front. Psychol.* **4**, 910 (2013).
S20. Fukui, H. & Yamashita, M. The effects of music and visual stress on testosterone and cortisol in men and women. *Neuro Endocrinol. Lett.* **24**, 173-180 (2003).
S21. Fusani, L., Metzdorf, R., Hutchison, J.B. & Gahr, M. Aromatase inhibition affects testosterone-induced masculinization of song and the neural song system in female canaries. *J. Neurobiol.* **54**, 370-379 (2003).
S22. Gerra, G. *et al.* Neuroendocrine responses of healthy volunteers to 'techno-music': relationships with personality traits and emotional state. *Int. J. Psychophysiol.* **28**, 99-111 (1998).
S23. Gervain, J. *et al.* Valproate reopens critical-period learning of absolute pitch. *Front. Syst. Neurosci.* **7**, 102 (2013).
S24. Gilbert, M.T. & Soderstrom, K. Novel song-stimulated dendritic spine formation and Arc/Arg3.1 expression in zebra finch auditory telencephalon are disrupted by cannabinoid agonism. *Brain Res.* **1541**, 9-21 (2013).
S25. Gingras, B., Pohler, G. & Fitch, W.T. Exploring shamanic journeying: repetitive drumming with shamanic instructions induces specific subjective experiences but no larger cortisol decrease than instrumental meditation music. *PLoS One* **9**, e102103 (2014).
S26. Granot, R.Y. *et al.* Provisional evidence that the arginine vasopressin 1a receptor gene is associated with musical memory. *Evol. Hum. Behav.* **28**, 313-318 (2007).
S27. Granot, R.Y., Uzefovsky, F., Bogopolsky, H. & Ebstein, R.P. Effects of arginine vasopressin on musical working memory. *Front. Psychol.* **4**, 712 (2013).
S28. Gregersen, P.K. *et al.* Absolute pitch exhibits phenotypic and genetic overlap with synesthesia. *Hum. Mol. Genet.* **22**, 2097-2104 (2013).
S29. Gunaratne, P.H. *et al.* Song exposure regulates known and novel microRNAs in the zebra finch auditory forebrain. *BMC Genomics* **12**, 277 (2011).
S30. Haesler, S. *et al.* FoxP2 expression in avian vocal learners and non-learners. *J. Neurosci.* **24**, 3164-3175 (2004).
S31. Haesler, S. *et al.* Incomplete and inaccurate vocal imitation after knockdown of FoxP2 in songbird basal ganglia nucleus Area X. *PLoS Biol.* **5**, e321 (2007).
S32. Hartog, T.E. *et al.* Brain-derived neurotrophic factor signaling in the HVC is required for testosterone-induced song of female canaries. *J. Neurosci.* **29**, 15511-15519 (2009).
S33. Hassler, M., Gupta, D. & Wollmann, H. Testosterone, estradiol, ACTH and musical, spatial and verbal performance. *Int. J. Neurosci.* **65**, 45-60 (1992).
S34. Heston, J.B. & White, S.A. Behavior-linked FoxP2 regulation enables zebra finch vocal learning. *J. Neurosci.* **35**, 2885-2894 (2015).
S35. Hilliard, A.T., Miller, J.E., Fraley, E.R., Horvath, S. & White, S.A. Molecular microcircuitry underlies functional specification in a basal ganglia circuit dedicated to vocal learning. *Neuron* **73**, 537-552 (2012).
S36. Huesmann, G.R. & Clayton, D.F. Dynamic role of postsynaptic caspase-3 and BIRC4 in zebra finch song-response habituation. *Neuron* **52**, 1061-1072 (2006).
S37. Jansen, R. *et al.* Melatonin affects the temporal organization of the song of the zebra finch. *FASEB J.* **19**, 848-850 (2005).
S38. Jarvis, E.D., Scharff, C., Grossman, M.R., Ramos, J.A. & Nottebohm, F. For whom the bird sings: context-dependent gene expression. *Neuron* **21**, 775-788 (1998).
S39. Kanduri, C. *et al.* The effect of music performance on the transcriptome of professional musicians. *Sci. Rep.* **5**, 9506 (2015).
S40. Kanduri, C. *et al.* The effect of listening to music on human transcriptome. *PeerJ* **3**, e830 (2015).
S41. Keeler, J.R. *et al.* The neurochemistry and social flow of singing: bonding and oxytocin.

Front. Hum. Neurosci. **9**, 518 (2015).

S42. Khalfa, S., Bella, S.D., Roy, M., Peretz, I. & Lupien, S.J. Effects of relaxing music on salivary cortisol level after psychological stress. *Ann. N.Y. Acad. Sci.* **999**, 374-376 (2003).

S43. Kimpo, R.R. & Doupe, A.J. FOS is induced by singing in distinct neuronal populations in a motor network. *Neuron* **18**, 315-325 (1997).

S44. Knight, W.E. & Rickard Ph, D.N. Relaxing music prevents stress-induced increases in subjective anxiety, systolic blood pressure, and heart rate in healthy males and females. *J. Music Ther.* **38**, 254-272 (2001).

S45. Kreutz, G., Bongard, S., Rohrmann, S., Hodapp, V. & Grebe, D. Effects of choir singing or listening on secretory immunoglobulin A, cortisol, and emotional state. *J. Behav. Med.* **27**, 623-635 (2004).

S46. Kurz, A. *et al.* Alpha-synuclein deficiency leads to increased glyoxalase I expression and glycation stress. *Cell. Mol. Life Sci.* **68**, 721-733 (2011).

S47. Kurz, A. *et al.* Alpha-synuclein deficiency affects brain Foxp1 expression and ultrasonic vocalization. *Neuroscience* **166**, 785-795 (2010).

S48. Lai, H.-L. & Li, Y.-M. The effect of music on biochemical markers and self-perceived stress among first-line nurses: a randomized controlled crossover trial. *J. Adv. Nurs.* **67**, 2414-2424 (2011).

S49. LeBlanc, M.M., Goode, C.T., MacDougall-Shackleton, E.A. & Maney, D.L. Estradiol modulates brainstem catecholaminergic cell groups and projections to the auditory forebrain in a female songbird. *Brain Res.* **1171**, 93-103 (2007).

S50. Leblois, A. & Perkel, D.J. Striatal dopamine modulates song spectral but not temporal features through D1 receptors. *Eur. J. Neurosci.* **35**, 1771-1781 (2012).

S51. Liu, X. *et al.* Detecting signatures of positive selection associated with musical aptitude in the human genome. *Sci. Rep.* **6**, 21198 (2016).

S52. Lombardino, A.J., Li, X.C., Hertel, M. & Nottebohm, F. Replaceable neurons and neurodegenerative disease share depressed UCHL1 levels. *P. Natl. Acad. Sci. U.S.A.* **102**, 8036-8041 (2005).

S53. London, S.E. & Clayton, D.F. Functional identification of sensory mechanisms required for developmental song learning. *Nat. Neurosci.* **11**, 579-586 (2008).

S54. Lynch, K.S., Diekamp, B. & Ball, G.F. Colocalization of immediate early genes in catecholamine cells after song exposure in female zebra finches (Taeniopygia guttata). *Brain Behav Evol* **79**, 252-260 (2012).

S55. Mangiamele, L.A. & Burmeister, S.S. Acoustically evoked immediate early gene expression in the pallium of female tungara frogs. *Brain Behav. Evol.* **72**, 239-250 (2008).

S56. Matsunaga, E., Suzuki, K., Kobayashi, T. & Okanoya, K. Comparative analysis of mineralocorticoid receptor expression among vocal learners (Bengalese finch and budgerigar) and non-vocal learners (quail and ring dove) has implications for the evolution of avian vocal learning. *Dev. Growth Differ.* **53**, 961-970 (2011).

S57. McCraty, R., Atkinson, M., Rein, G. & Watkins, A.D. Music enhances the effect of positive emotional states on salivary IgA. *Stress Medicine* **12**, 167-175 (1996).

S58. Mello, C.V., Vicario, D.S. & Clayton, D.F. Song presentation induces gene expression in the songbird forebrain. *P. Natl. Acad. Sci. U.S.A.* **89**, 6818-6822 (1992).

S59. Meng, B., Zhu, S., Li, S., Zeng, Q. & Mei, B. Global view of the mechanisms of improved learning and memory capability in mice with music-exposure by microarray. *Brain Res. Bull.* **80**, 36-44 (2009).

S60. Merullo, D.P., Cordes, M.A., DeVries, S.M., Stevenson, S.A. & Riters, L.V. Neurotensin neural mRNA expression correlates with vocal communication and other highly-motivated social behaviors in male European starlings. *Physiol. Behav.* **151**, 155-161 (2015).

S61. Miller, J.E., Hafzalla, G.W., Burkett, Z.D., Fox, C.M. & White, S.A. Reduced vocal variability in a zebra finch model of dopamine depletion: implications for Parkinson disease. *Physiol. Rep.* **3**, e12599 (2015).

S62. Miller, J.E. *et al.* Birdsong decreases protein levels of FoxP2, a molecule required for human speech. *J. Neurophysiol.* **100**, 2015-2025 (2008).
S63. Möckel, M. *et al.* [Stress reduction through listening to music: effects on stress hormones, hemodynamics and mental state in patients with arterial hypertension and in healthy persons]. *Dtsch. Med. Wochenschr.* **120**, 745-752 (1995).
S64. Monbureau, M., Barker, J.M., Leboucher, G. & Balthazart, J. Male song quality modulates c-Fos expression in the auditory forebrain of the female canary. *Physiol. Behav.* **147**, 7-15 (2015).
S65. Mori, C. & Wada, K. Audition-independent vocal crystallization associated with intrinsic developmental gene expression dynamics. *J. Neurosci.* **35**, 878-889 (2015).
S66. Morley, A.P. *et al.* AVPR1A and SLC6A4 polymorphisms in choral singers and non-musicians: a gene association study. *PLoS One* **7**, e31763 (2012).
S67. Murugan, M., Harward, S., Scharff, C. & Mooney, R. Diminished FoxP2 levels affect dopaminergic modulation of corticostriatal signaling important to song variability. *Neuron* **80**, 1464-1476 (2013).
S68. Nastiuk, K.L., Mello, C.V., George, J.M. & Clayton, D.F. Immediate-early gene responses in the avian song control system: cloning and expression analysis of the canary c-jun cDNA. *Brain Res. Mol. Brain Res.* **27**, 299-309 (1994).
S69. Nilsson, U. Soothing music can increase oxytocin levels during bed rest after open-heart surgery: a randomised control trial. *J. Clin. Nurs.* **18**, 2153-2161 (2009).
S70. Oikkonen, J. *et al.* A genome-wide linkage and association study of musical aptitude identifies loci containing genes related to inner ear development and neurocognitive functions. *Mol. Psychiatr.* **20**, 275-282 (2015).
S71. Olson, C.R., Hodges, L.K. & Mello, C.V. Dynamic gene expression in the song system of zebra finches during the song learning period. *Dev. Neurobiol.* **75**, 1315-1338 (2015).
S72. Park, H. *et al.* Comprehensive genomic analyses associate UGT8 variants with musical ability in a Mongolian population. *J. Med. Genet.* **49**, 747-752 (2012).
S73. Pinaud, R., C, O., O, A. & Ed, J. Profiling of experience-regulated proteins in the songbird auditory forebrain using quantitative proteomics. *Eur. J. Neurosci.* **27**, 1409-1422 (2008).
S74. Poopatanapong, A. *et al.* Singing, but not seizure, induces synaptotagmin IV in zebra finch song circuit. *J. Neurobiol.* **66**, 1613-1629 (2006).
S75. Qu, S., Olafsrud, S.M., Meza-Zepeda, L.A. & Saatcioglu, F. Rapid gene expression changes in peripheral blood lymphocytes upon practice of a comprehensive yoga program. *PLoS One* **8**, e61910 (2013).
S76. Rauceo, S. *et al.* Dopaminergic modulation of reproductive behavior and activity in male zebra finches. *Behav. Brain. Res.* **187**, 133-139 (2008).
S77. Ringel, L.E., Basken, J.N., Grant, L.M. & Ciucci, M.R. Dopamine D1 and D2 receptor antagonism effects on rat ultrasonic vocalizations. *Behav. Brain. Res.* **252**, 252-259 (2013).
S78. Riters, L.V., Stevenson, S.A., DeVries, M.S. & Cordes, M.A. Reward associated with singing behavior correlates with opioid-related gene expression in the medial preoptic nucleus in male European starlings. *PLoS One* **9**, e115285 (2014).
S79. Salimpoor, V.N., Benovoy, M., Larcher, K., Dagher, A. & Zatorre, R.J. Anatomically distinct dopamine release during anticipation and experience of peak emotion to music. *Nat. Neurosci.* **14**, 257-262 (2011).
S80. Sanyal, T. *et al.* Prenatal loud music and noise: differential impact on physiological arousal, hippocampal synaptogenesis and spatial behavior in one day-old chicks. *PLoS One* **8**, e67347 (2013).
S81. Sasaki, A., Sotnikova, T.D., Gainetdinov, R.R. & Jarvis, E.D. Social context-dependent singing-regulated dopamine. *J. Neurosci.* **26**, 9010-9014 (2006).
S82. Schwilling, D. *et al.* Live music reduces stress levels in very low-birthweight infants. *Acta Paediatr.* **104**, 360-367 (2015).
S83. Shi, Z. *et al.* miR-9 and miR-140-5p target FoxP2 and are regulated as a function of the

social context of singing behavior in zebra finches. *J. Neurosci.* **33**, 16510--16521 (2013).
S84. Shu, W. *et al.* Altered ultrasonic vocalization in mice with a disruption in the Foxp2 gene. *P. Natl. Acad. Sci. U.S.A.* **102**, 9643-9648 (2005).
S85. Sia, G.M., Clem, R.L. & Huganir, R.L. The human language-associated gene SRPX2 regulates synapse formation and vocalization in mice. *Science* **342**, 987-991 (2013).
S86. Singh, T., Basham, M., Nordeen, E. & Nordeen, K. Early sensory and hormonal experience modulate age-related changes in NR2B mRNA within a forebrain region controlling avian vocal learning. *J. neurobiol.* **44**, 82-94 (2000).
S87. Stefano, G.B., Zhu, W., Cadet, P., Salamon, E. & Mantione, K.J. Music alters constitutively expressed opiate and cytokine processes in listeners. *Med. Sci. Monit.* **10**, MS18-27 (2004).
S88. Sutoo, D. & Akiyama, K. Music improves dopaminergic neurotransmission: demonstration based on the effect of music on blood pressure regulation. *Brain Res.* **1016**, 255-262 (2004).
S89. Teramitsu, I., Poopatanapong, A., Torrisi, S. & White, S.A. Striatal FoxP2 is actively regulated during songbird sensorimotor learning. *PLoS One* **5**, e8548 (2010).
S90. Teramitsu, I. & White, S.A. FoxP2 regulation during undirected singing in adult songbirds. *J. Neurosci.* **26**, 7390-7394 (2006).
S91. Theusch, E., Basu, A. & Gitschier, J. Genome-wide study of families with absolute pitch reveals linkage to 8q24.21 and locus heterogeneity. *Am. J. Hum. Genet.* **85**, 112-119 (2009).
S92. Thoma, M.V. *et al.* The effect of music on the human stress response. *PLoS One* **8**, e70156 (2013).
S93. Thompson, C.K. *et al.* Young and intense: FoxP2 immunoreactivity in Area X varies with age, song stereotypy, and singing in male zebra finches. *Front. Neural Circuits* **7**, 24 (2013).
S94. Tremere, L.A., Jeong, J.K. & Pinaud, R. Estradiol shapes auditory processing in the adult brain by regulating inhibitory transmission and plasticity-associated gene expression. *J. Neurosci.* **29**, 5949-5963 (2009).
S95. Ukkola, L.T., Onkamo, P., Raijas, P., Karma, K. & Järvelä, I. Musical aptitude is associated with AVPR1A-haplotypes. *PLoS One* **4**, e5534 (2009).
S96. VanderArk, S.D. & Ely, D. Cortisol, biochemical, and galvanic skin responses to music stimuli of different preference values by college students in biology and music. *Percept. Mot. Skills* **77**, 227-234 (1993).
S97. Velho, T. A., Pinaud, R., Rodrigues, P.V., & Mello, C.V. Co-induction of activity-dependent genes in songbirds. *Eur. J. Neurosci.* **22**, 1667-1678 (2005).
S98. Velho, T.A. *et al.* Noradrenergic control of gene expression and long-term neuronal adaptation evoked by learned vocalizations in songbirds. *PLoS One* **7**, e36276 (2012).
S99. Velho, T.A. & Mello, C.V. Synapsins are late activity-induced genes regulated by birdsong. *J. Neurosci.* **28**, 11871-11882 (2008).
S100. Wada, K. *et al.* A molecular neuroethological approach for identifying and characterizing a cascade of behaviorally regulated genes. *P. Natl. Acad. Sci. U.S.A.* **103**, 15212-15217 (2006).
S101. Warren, W.C. *et al.* The genome of a songbird. *Nature* **464**, 757-762 (2010).
S102. Whitney, O. *et al.* Core and region-enriched networks of behaviorally regulated genes and the singing genome. *Science* **346**, 1256780 (2014).
S103. Xu, J., Yu, L., Cai, R., Zhang, J. & Sun, X. Early auditory enrichment with music enhances auditory discrimination learning and alters NR2B protein expression in rat auditory cortex. *Behav. Brain. Res.* **196**, 49-54 (2009).
S104. Yang, E.J., Lin, E.W. & Hensch, T.K. Critical period for acoustic preference in mice. *P. Natl. Acad. Sci. U.S.A.* **109** Suppl 2, 17213-17220 (2012).
S105. Yoder, K.M., Phan, M.L., Lu, K. & Vicario, D.S. He hears, she hears: are there sex differences in auditory processing? *Dev. Neurobiol.* **75**, 302-314 (2015).
